# The Accuracy of Empirical Antibiotic Treatment for Periprosthetic Joint Infections in Total Shoulder and Knee Arthroplasties

**DOI:** 10.3390/antibiotics14050447

**Published:** 2025-04-28

**Authors:** Katrin Freller, Hannah Punz, Clemens Schopper, Tobias Gotterbarm, Antonio Klasan, Stella Stevoska

**Affiliations:** 1 Faculty of Medicine, Johannes Kepler University Linz, 4040 Linz, Austria; hannah.punz@gmx.net (H.P.); clemens.schopper@kepleruniklinikum.at (C.S.); tobias.gotterbarm@kepleruniklinikum.at (T.G.); klasan.antonio@me.com (A.K.); 2 Department for Orthopedics and Traumatology, Kepler University Hospital GmbH, 4020 Linz, Austria; 3 AUVA UKH Steiermark, 8020 Graz, Austria

**Keywords:** empirical antibiotic, knee arthroplasty, shoulder arthroplasty, periprosthetic joint infections, revision, antibiotic resistance

## Abstract

**Introduction:** Periprosthetic joint infections (PJIs) remain a major challenge in orthopedic and trauma surgeries. The microbial resistance profiles and the optimal choice of empirical antibiotic therapy in shoulder arthroplasty revision are less well characterized compared to those in knee or hip arthroplasty revision. **Materials and Methods:** This retrospective study constitutes a novel comparative analysis, providing valuable insights into the presence of joint-specific pathogen resistance and the empirical treatment accuracy of shoulder and knee arthroplasties. A review of all the revision cases following primary shoulder and knee arthroplasties conducted between January 2012 and December 2023 was performed. Cases that required revision because of PJIs were identified, and microbial cultures were analyzed to determine the presence of pathogens and their resistance profiles. **Results:** The most administered postoperative empirical antibiotics were cefuroxime and amoxicillin–sulbactam. A statistically significant difference in the prevalence of anerobic pathogens was observed between total shoulder arthroplasty and knee arthroplasty. Furthermore, a statistically significant difference was observed in the sensitivities of pathogens to metronidazole (*p* < 0.001) and erythromycin (*p* = 0.014). **Conclusions:** This study demonstrates microbiological and antimicrobial resistance differences between PJI TSA and TKA cases.

## 1. Introduction

Periprosthetic joint infections (PJIs) represent a substantial challenge in orthopedic and trauma surgery. The incidence of PJIs in shoulder prostheses ranges from 0.7% to 3.9%, while in knee prostheses, it falls between 0.8% and 1.9% [1,2,3]. The predominant empirical antibiotic protocols are mainly based on broad-spectrum antibiotics, such as ampicillin/sulbactam or cefuroxime, until the availability of pathogen-specific data allows for a more targeted approach. It is of the utmost importance to select an appropriate empirical primary antibiotic to reduce the incidence of an antimicrobial mismatch or the failure of antibiotics to target the causal microorganisms responsible for PJIs [4]. The efficacy of empirical antibiotic therapy and the microbial antimicrobial resistance profiles in shoulder arthroplasty (TSA) remain poorly understood compared to those in knee arthroplasty (TKA) [5,6]. Direct comparative data on empirical antibiotic accuracy between TSA and TKA are limited, to the best of our knowledge. There have only been several studies comparing TKA and total hip arthroplasty (THA) revisions [7,8,9,10].

This study aims to evaluate the accuracy of empirical antibiotic therapy and compare the microbial resistance spectra between shoulder and knee arthroplasty revisions in cases of PJIs. Furthermore, it seeks to ascertain pathogen resistance to facilitate more informed guidance for clinical practice.

## 2. Results

**Figure 1:**
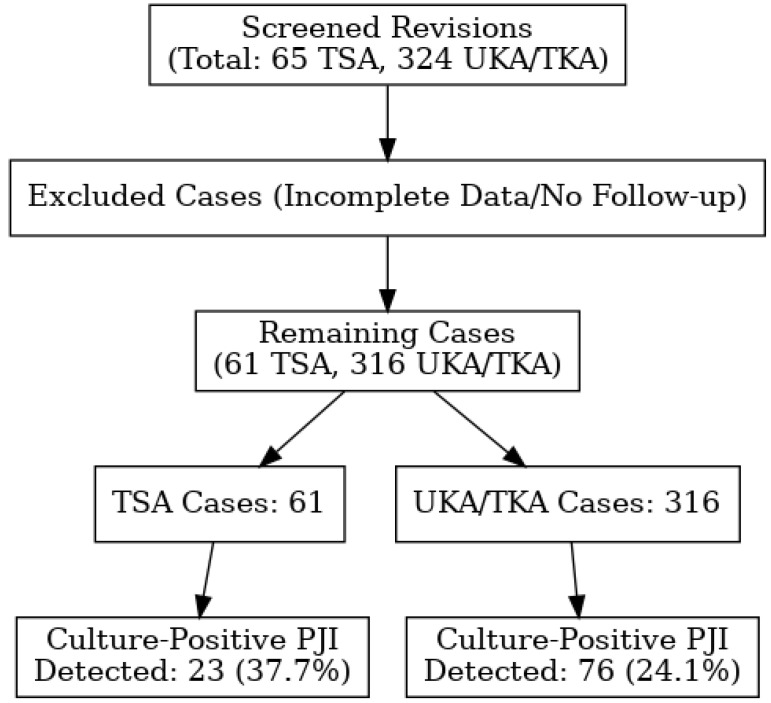
Flowchart of patients and culture inclusion criteria.

A total of 65 total shoulder arthroplasty (TSA) revisions and 324 unicompartmental or total knee arthroplasty (UKA/TKA) revisions, performed between 2012 and 2023, were screened for inclusion in this study (Figure 1). Cases with incomplete data or no follow-up were excluded, leaving 61 TSA revisions and 316 TKA/UKA revisions for further evaluation. In the shoulder arthroplasty revision group, culture-positive PJIs were detected in 23 out of 61 revision cases (37.7%). In the knee arthroplasty revision group, culture-positive PJIs were identified in 76 out of 316 revision cases (24.1%).

### 2.1. Shoulder Arthroplasty Revision Group

A total of fifteen male and eight female patients who had undergone TSA revision because of PJIs were included in this study. The mean age of the patients was 67.6 (ranging from 49 to 84) years, with a mean body mass index (BMI) of 27.7 kg/m^2^. The patients were classified according to the American Society of Anesthesiologists’ (ASA) score as follows: zero patients were classified as ASA-1, fifteen patients as ASA-2, eight patients as ASA-3, and zero patients as ASA-4. The demographics are visualized in Table 1.

Cefuroxime was the most frequently utilized empirical antibiotic in TSA, used in eight out of twenty-three cases (34.8%), followed by clindamycin, which was used in five out of twenty-three cases (21.7%). The remaining antibiotics utilized were ampicillin/sulbactam, penicillin G, amoxicillin/clavulanic acid, and vancomycin (Table 2).

### 2.2. Knee Arthroplasty Revision Group

A total of 38 male and 38 female patients, who had undergone TKA or UKA revision because of PJIs, were included in this study. The mean age of the patients was 70.2 (ranging from 51 to 93) years, with a mean BMI of 32.6 kg/m^2^. The patients were classified according to their ASA score as follows: five patients were classified as ASA-1, thirty-seven patients as ASA-2, thirty-three patients as ASA-3, and one patient as ASA-4 (Table 1).

In the empirical administration of antibiotics in knee arthroplasty revision, ampicillin/sulbactam was the most prevalent agent, used in 40 out of 76 cases (52.6%), followed by cefuroxime, which was utilized in 21 out of 76 cases (27.6%) (Table 2).

### 2.3. Pathogen Identification

In shoulder arthroplasty revision, polymicrobial infections were identified in three patients, while in knee arthroplasty revision, multi-pathogen infections were identified in four patients. A total of 27 bacterial strains were isolated in TSA, while 83 bacterial strains were identified in TKA/UKA. Coagulase-negative *staphylococci* (CNS) were the most common group of bacteria—in 59.3% (16 out of 27) of the cultures after TSA revision and in 43.4% (36 out of 830) of the cultures after TKA/UKA revision (Table 3). *Staphylococcus epidermidis* was the most common type of CNS and the most common type of bacteria in TSA revision and TKA/UKA revision. In TSA revision, 44.4% (12 out of 27 cultures) of the samples had *Staphylococcus epidermidis*. In the TKA/UKA group, *Staphylococcus epidermidis* was identified in 25.3% of the cases (21 out of 83 cultures).

Notably, in TKA/UKA, *Staphylococcus aureus* constituted the second-most prevalent bacteria (24.1%, twenty out of eighty-three cultures), while in TSA, anerobic bacteria—*Cutibacterium acnes*, were the second-most prevalent (25.9%, seven out of twenty-seven). There were no Gram-negative bacteria detected in the TSA group, whereas in the TKA/UKA group, Gram-negative strains were isolated (6.0%, five out of eighty-three cultures). One strain of *methicillin-resistant Staphylococcus aureus (MRSA)* was identified in the TKA/UKA group, and none were identified in the TSA group. No Streptococcus spp. was identified in the TSA group, whereas in the TKA/UKA group, 15.7% (13 out of 83 cultures) of all the identified bacteria were *Streptococcus* spp., representing a statistically significant difference in prevalence (*p* = 0.029). A statistically significant difference was also observed in the prevalence of anerobic bacteria between TSA (25.9%) and TKA/UKA (3.6%) cultures (*p* < 0.001). The most prevalent anerobe identified in TSA cases was *Cutibacterium acnes* (25.9%). Enterococcus spp. was isolated at rates of 3.7% in TSA and 7.2% in TKA/UKA, with *Enterococcus faecalis* being more prevalent in TKA/UKA (4.8%). Gram-negative bacteria were identified exclusively in TKA cases (6.0%). However, the difference between those groups was not statistically significant.

### 2.4. Empirical Therapy Accuracy

**Figure 2:**
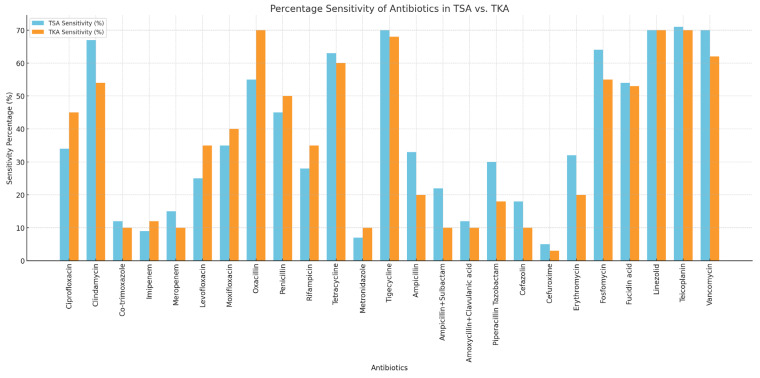
Sensitivity of antibiotics in TSA vs. TKA.

The occurrence of resistance in patients who had undergone TKA/UKA and TSA revisions was analyzed for a total of 25 different antibiotics, including ciprofloxacin, clindamycin, co-trimoxazole, imipenem, meropenem, levofloxacin, moxifloxacin, oxacillin, penicillin, rifampicin, tetracycline, metronidazole, tigecycline, ampicillin, ampicillin/sulbactam, amoxicillin/clavulanic acid, piperacillin/tazobactam, cefazolin, cefuroxime, erythromycin, fosfomycin, fusidic acid, linezolid, teicoplanin, and vancomycin (Figure 2).

The complete antibiogram showed that several antibiotics were sensitive in the TSA and TKA/UKA groups. Both groups maintained full sensitivity to linezolid, teicoplanin, meropenem, rifampicin, and vancomycin. In the TSA revision group, full sensitivity was also observed for imipenem, tetracycline, tigecycline, amoxicillin/clavulanic acid, and cefuroxime. Additionally, in the TSA group, co-trimoxazole (17/18 strains, 94.4%) and fosfomycin (18/19 strains, 94.7%) exhibited high sensitivity rates. In the TKA/UKA group, co-trimoxazole (41/45 strains, 91.1%), tetracycline (49/53 strains, 92.5%), and tigecycline (54/55 strains, 98.2%) demonstrated high sensitivity rates. The sensitivity comparison between TSA and TKA/UKA is shown in Figure 2.

A comparison of the sensitivities and resistance rates in the two groups revealed two significant differences. Significant differences were observed in the sensitivities of metronidazole (*p* < 0.001) and erythromycin (*p* = 0.014) (Table 4). Metronidazole exhibited a statistically significant discrepancy in its resistance patterns, with 100.0% resistance recorded in the TSA group and only a 20.0% resistance rate observed in the TKA/UKA species (*p* < 0.001). However, no significant differences were noted in the resistances of the strains to the other antibiotics. The highest resistance rates were observed in TSA for metronidazole (100.0%) and ampicillin (100.0%). In TKA/UKA, penicillin (thirty-two/sixty strains, 53.3%) and ampicillin (eight/sixteen strains, 50.0%) had the highest resistance rates.

The overall accuracy of the empirical antibiotics was not significantly different between shoulder and knee arthroplasty revisions. The accuracy was found to be 70.4% for the TSA revision and 75.9% for the TKA/UKA revision groups. The most prescribed empirical antibiotics in the TSA revision group were cefuroxime, ampicillin/sulbactam, and clindamycin. Clindamycin, despite being the second most used empirical agent in TSA revisions (21.7%), exhibited only 33.3% accuracy (two out of six isolates were sensitive) in the TSA group and 60.0% in the TKA/UKA group (Table 5).

## 3. Discussion

This study presents a methodical and comprehensive comparison of the microbiological profiles and antibiotic resistance patterns between TKA and TSA revisions. The study observed significant differences in bacterial diversity, the prevalence of pathogens, and antimicrobial susceptibility profiles between the two surgical sites, which are critical for clinical management and infection control strategies. Nevertheless, the overall accuracy of the empirical antibiotics was not significantly different between shoulder and knee arthroplasty revisions.

### 3.1. Pathogens

The analysis revealed the predominance of Gram-positive bacteria in both TSA and TKA/UKA cultures, with a notable isolation of coagulase-negative *staphylococci* (CNS) in both groups. The present study is consistent with the findings of recent studies [9,11,12], which determined that the most prevalent pathogen in Europe was CNS. In our data, the prevalence of CNS was higher in TSA (59.3%) than in TKA (43.4%); however, the difference was not statistically significant (*p* = 0.15).

Several studies have indicated that in cases of shoulder PJIs, the most prevalent pathogens are *Cutibacterium acnes* and *Staphylococcus aureus* [13,14,15,16,17]. A recent study by Nelson et al. [5] reported that *C. acnes* constituted the most frequently identified pathogen (38.9%) in PJIs of shoulder arthroplasty. Studies by Tande et al. [18] and Bdeir et al. [19] reported similar findings. In contrast to the studies mentioned above, in the present study of shoulder PJIs, *Cutibacterium acnes* (*n* = 7; 25.9%) was only the second most prevalent pathogen, while *Staphylococcus epidermidis* (*n* = 12; 44.4%) was the most identified pathogen. The data show a higher prevalence of anerobic bacteria in TSA, especially *C. acnes* (25.9% vs. 3.6%, *p* < 0.001), which indicates differences in skin flora and surgical exposure. This finding is of clinical significance, suggesting the importance of anerobic coverage in empirically based regimens for TSA PJIs. *C. acnes* can often lead to diagnostic challenges because of its gradual progression and required prolonged cultivation period [13,14,18]; however, our cultivation period of 14 days should be sufficient to detect any bacterial growth that might occur, even in slower-growing or less viable specimens. Nevertheless, the occurrence of *Cutibacterium acnes* in the present study was statistically significantly higher in shoulder arthroplasty compared to knee arthroplasty (*p* < 0.001). On the other hand, a statistically significantly higher rate of Streptococcus spp. was observed in TKA/UKA cases (*p* = 0.029), consistent with the findings of Nodzo et al.’s [15] study. Nodzo et al. [15] also highlighted the increased presence of Enterococcus spp. and Gram-negative bacteria in TKA compared to TSA. These findings were similar to the results of our observation, where *Enterococcus* spp. were identified in 7.2% of the TKA/UKA cases. Additionally, Gram-negative bacteria were exclusively present in TKA/UKA (6.0%) and absent in TSA. However, the differences in these rates compared to those in shoulder arthroplasty were not statistically significant (*p* = 0.51 and *p* = 0.64). The observed prevalence of methicillin-resistant and Gram-negative organisms corresponded to the regional antimicrobial surveillance data documented in the *Austrian Resistance Report* (AURES) [20].

Overall, TKA/UKA cultures showed a higher diversity of bacterial species, comprising streptococci and Gram-negative bacteria. The management of PJIs caused by Gram-negative pathogens is a more challenging clinical problem and has been associated with limited success in treatment outcomes [21,22].

### 3.2. Antibiotic Resistance and Empirical Antibiotics

Currently, there is no universally accepted standard for the empirical antibiotic treatment of PJIs. The predominant strategy involves the empirical administration of ampicillin/sulbactam, which aligns with our findings. Bdeir et al. reported clindamycin as the most frequently employed empirical antibiotic in shoulder PJIs [23]. However, rising resistance rates against clindamycin may limit its efficacy [24]. Van Erp et al. [25] suggest cefazolin as an empirical antibiotic in early PJIs. A few studies suggest using vancomycin or daptomycin, which are effective against methicillin-resistant Gram-positive cocci [11,26,27,28]. The low rate of MRSA (1.2%) observed in this study suggests that routine vancomycin coverage may not be warranted. However, there was a quite-high rate of methicillin-resistant CNS in shoulder (36.8%) and knee (30.2%) arthroplasties (*p* = 0.540). These findings emphasize the need for ongoing surveillance of resistance patterns and potential adjustments to empirical antibiotic strategies if resistance rates continue to rise.

The significantly higher prevalence of *C. acnes* justifies anerobic coverage for TSA, whereas broader inclusion of Streptococcus spp. and Gram-negative pathogens could be considered for TKA. *C. acnes* is typically susceptible to narrow-spectrum agents, such as penicillin and amoxicillin [24]. Therefore, the empirical use of ampicillin/sulbactam seems to be an appropriate choice for shoulder arthroplasty infections. Although there was a higher rate of Gram-negative pathogens isolated in knee PJIs, this was not significantly higher compared to the rate in shoulder PJI cases (*p* = 0.640). Moreover, this did not impact the accuracy of the empirical antibiotic selection. The overall accuracy rates were comparable between the groups, with 70.4% for the TSA revision group and 75.9% for the TKA/UKA revision group (*p* = 0.200).

The present study showed the high resistance rates of pathogens to clindamycin, leading to a low accuracy rate in its empirical use—only 33.3% in the TSA group and 60.0% in the TKA/UKA group. Clindamycin was used in multiple cases because of reported penicillin allergies. Unfortunately, preoperative allergy testing was not conducted to confirm the specific nature of these reported allergies. Clindamycin was mainly chosen by surgeons as an alternative empirical antibiotic in these cases. However, the limited accuracy observed in the available data highlights the ongoing need for the reevaluation of these empirical decisions. Given the preserved susceptibility of pathogens to vancomycin, it appears that vancomycin is a more reliable option for empirical antibiotic therapy in cases of penicillin allergy. It is reassuring that both groups showed no resistance to key antibiotics, such as linezolid, teicoplanin, rifampicin, and vancomycin, which are very important agents in the treatment of Gram-positive PJIs. Co-trimoxazole and fosfomycin were also found to be highly susceptible (>90.0%) in both groups, supporting their use for the targeted therapy of staphylococcal infections.

There was a statistically significant difference in metronidazole (*p* < 0.001) and erythromycin (*p* = 0.014) resistances between the two groups because of site-specific microbiological profiles. The predominant metronidazole resistance in TSA is consistent with the dominance of anerobic bacteria, particularly *C. acnes*, which is inherently resistant to this antibiotic [29]. Although metronidazole displayed 100% resistance in the context of TSA, its clinical relevance is constrained because of its exclusion from standard empirical protocols for the management of TSA PJIs. However, despite differences in microbiological characteristics and antibiotic resistance, these factors did not affect the accuracy of the empirical antibiotic selection between shoulder and knee PJIs (*p* = 0.200).

Despite these insights, this study has certain limitations, including a lack of data on patient comorbidities or surgical techniques, all of which could influence infection patterns and treatment responses. The dataset did not consistently record ethnicity; therefore, it could not be included in the demographic analysis. However, given that this study was conducted in Europe, the sample likely comprised predominantly Caucasian participants. Although our findings indicate a relatively high accuracy rate for the most common preoperative empirical antibiotics, cefuroxime and ampicillin/sulbactam, certain trends have emerged that merit careful consideration. For example, the presence of anerobic pathogens, such as Cutibacterium acnes, in TSA revisions could support the use of empirical antibiotics with improved anerobic coverage in particular cases. However, because of the retrospective and single-center nature of this study, it is not possible to recommend definitive changes to empirical protocols. False-negative and false-positive rates in culture-based diagnosis are acknowledged limitations in PJI studies, particularly in the context of low-virulence organisms. However, the use of extended incubation and sonication was employed as a method to reduce such diagnostic errors. When analyzing low *n*-numbers, it is essential to exercise caution in interpretation. The sample size for TSA was relatively small (*n* = 61), which may reduce the statistical power for subgroup comparisons and limit the broader applicability of these results. Despite the consistent implementation of microbiological protocols throughout the study period, changes in resistance patterns or surgical methods over time may have influenced the results. Another potential limitation to consider is that the surveillance data over the 14-year period were not analyzed by time stratification. This limitation makes it challenging to interpret potential temporal trends. This study was not provided with data on MRSA colonization and prior outpatient antibiotic use, which could influence resistance patterns and introduce selection bias. Additionally, the retrospective single-center design introduces the potential for selection bias. Future multi-center studies with larger cohort sizes and standardized sampling techniques will be essential for confirming these observations and generating more generalizable findings.

## 4. Materials and Methods

A retrospective analysis was conducted on all the patients who underwent arthroplasty revision, either total shoulder arthroplasty or total knee arthroplasty, between January 2012 and December 2023. The study was approved by the University’s Ethics Board (Approval Number: 1116/2024). It included all the cases that met the consensus criteria for PJIs, in which an organism had been identified [30]. Cases with insufficient data were excluded from the study. All the microbiological samples were processed at the same institutional microbiology laboratory using standardized protocols, ensuring consistency across all the cases.

All the antibiotic dosages and formulations used for antibiogram testing were based on the guidelines of the European Committee on Antimicrobial Susceptibility Testing (EUCAST). In the context of periprosthetic joint infections (PJIs), high-dose regimens are standard in clinical practice to ensure adequate tissue penetration and therapeutic efficacy. Therefore, for all 25 antibiotics listed in Figure 2, the high-dose recommendations according to EUCAST were consistently applied [31]. The dataset was collected from electronic medical records, outpatient charts, and surgical reports that had been previously documented. Moreover, in case of PJIs, additional parameters were integrated into the dataset by collecting and analyzing existing microbiological testing and antibiograms. All the findings with bacterial evidence were considered as relevant. The accuracy of the empirical antibiotic therapy was defined as the proportion of cases in which the initial antibiotic administered showed in vitro sensitivity against the isolated pathogens. This definition was applied in the same way to both the TSA and TKA/UKA groups so that the results could be directly compared.

### 4.1. Surgical Treatment Protocol

There were no changes in the preoperative washing protocol, skin preparation method, hand hygiene solutions, or sterilization procedures for surgical equipment, nor were there any significant differences in the surgical approach during the study period. MRSA colonization status was not evaluated. All the patients presenting with potential PJIs, as indicated by elevated C-reactive protein (CRP) levels and/or signs of local inflammation, underwent sterile aspiration of the synovial fluid and microbiological analysis. According to the timing of the infection and clinical findings, patients received one of the following treatments:

- Debridement and implant retention (DAIR);
- One-stage arthroplasty revision;
- Two-stage arthroplasty revision.

These interventions were followed by long-term antibiotic suppression. The revision techniques for PJIs remained consistent throughout the study period.

### 4.2. Prophylactic Antibiotics

In shoulder arthroplasty revisions, a preoperative single dosage of cefuroxime was used in twenty out of twenty-three patients (87.0%) and clindamycin in three out of twenty-three patients (13.0%) (Table 6).

In the knee arthroplasty revision group, 67 out of 76 patients (88.2%) received cefuroxime, among the preoperative single-shot medications utilized. Subsequently, clindamycin was administered to seven out of seventy-six patients (9.2%), one out of seventy-six patients (1.3%) received ampicillin/sulbactam, and in one out of seventy-six cases (1.3%), vancomycin was administered. The data are presented in Table 7.

### 4.3. Empirical Antibiotic Treatment

Revision surgery was followed by the administration of standardized empirical intravenous antibiotic regimens, typically targeting a broad spectrum of potential pathogens. Empirical therapy was continued until the availability of the microbiological identification of the causative organism and the corresponding antibiogram. Once these elements were obtained, targeted treatment adjustments were implemented.

### 4.4. Microbiology

The diagnoses of all the cases of PJIs were made in accordance with the criteria established by the Musculoskeletal Infection Society (MSIS), as reported by Parvizi et al. [30]. In cases of suspected PJIs, a routine preoperative sterile aspiration of the synovial fluid was performed, and at least three tissue samples and one sample of synovial fluid were obtained for culture intraoperatively.

The synovial fluid was aseptically inoculated in both aerobic and anerobic blood culture bottles (BACTEC, Beckton Dickinson, Franklin Lakes, NJ, USA) and incubated for 14 days. Tissue samples were collected from subfascial tissue and the proximal and distal interfaces of the prosthesis. Each culture was transported, processed, and analyzed according to international standards and the definitions of the European Committee on Antimicrobial Susceptibility Testing (EUCAST). The tissue samples were inoculated on Columbia agar, chocolate agar, McConkey agar, and Schädler agar and in brain–heart infusion (BHI) broth. The broth was subcultured the next day. Additionally, sonication of the explanted prosthetic components was performed. The supernatant was aspirated, and the sediment was inoculated in BHI broth. All the culture media were incubated for 14 days and inspected every day for bacterial growth. There were no major differences in tissue sampling between shoulder and knee arthroplasties over time.

Oxacillin was used as a reference to determine resistance within the penicillin and cephalosporin antibiotic groups.

#### 4.4.1. Outcome Measures

The primary outcome measure of this retrospective study was to identify and compare the isolated pathogens and their antimicrobial resistances between shoulder and knee arthroplasty revisions. In addition, secondary outcome measures encompassed the accuracy of the empirical antibiotic therapy between the two groups.

#### 4.4.2. Statistical Analysis

The data were collected using Microsoft Excel 16.96 (Redmond, WA, USA) and analyzed with SPSS v. 24 (IBM, Armonk, NY, USA). The study cohort was described using descriptive statistics as follows:

- Categorical variables were reported as percentages of the total sample for those variables;
- Continuous variables were expressed as means and standard deviations, and follow-up times were expressed as medians and interquartile ranges.

A post hoc power analysis was not conducted because of the inclusion of consecutive patients and demonstrated significance [32]. Overall antibiotic resistance was calculated by determining the number of antibiotics to which each culture was sensitive. For categorical variables, the differences between the cohorts were analyzed using the chi-squared test. For continuous variables with a normal distribution, a *t*-test was used; for a non-normal distribution, the Mann–Whitney U test was used. A post hoc power analysis was conducted to assess non-inferiority between the groups. The overall antibiotic resistance was calculated by determining the number of antibiotics to which each culture was sensitive. The presence of an interaction and the roles of confounding factors were evaluated. A *p*-value of <0.05 was considered as statistically significant.

## 5. Conclusions

This study demonstrates microbiological and antimicrobial resistance differences between PJI TSA and TKA cases. However, despite differences in microbiological characteristics and antibiotic resistances, these factors did not significantly affect the accuracy of the empirical antibiotic selection between shoulder and knee PJIs. Further research should focus on larger populations to confirm these findings and to optimize empirical protocols for PJIs.

## Figures and Tables

**Table 1:** Demographics in shoulder and knee arthroplasty revisions.

Variable	TSA (*n* = 23)	TKA/UKA (*n* = 76)
Mean age, years (SD)	67.56 (9.9)	70.23 (11.0)
Gender, *n* (%):		
Female	8 (35.0)	38 (50.0)
Male	15 (65.0)	38 (50.0)
Laterality, *n* (%):		
Left	9 (39.1)	40 (52.6)
Right	14 (60.7)	36 (47.4)
Arthroplasty, *n* (%)		
Explantation	10 (43.5)	8 (11.0)
One-stage prosthesis change	9 (39.1)	0 (0.0)
Inlay change	4 (17.4)	68 (89.0)
Mean BMI, kg/m^2^ (SD)	27.74 (4.3)	32.63 (7.8)
Mean ASA score	2.4	2.4
ASA score, *n* (%):		
I	0 (0.0)	5 (7.0)
II	15 (65.0)	37 (49.0)
III	8 (35.0)	33 (43.0)
IV	0 (0.0)	1 (1.0)

TSA, total shoulder arthroplasty; TKA, total knee arthroplasty; BMI, body mass index; ASA, American Society of Anesthesiologists; SD, standard deviation.

**Table 2:** Empirical antibiotics used because of PJIs in shoulder and knee arthroplasty revisions.

Empirical Antibiotic	Shoulder—No. of Patients, *n* (%)	Knee—No. of Patients, *n* (%)
Ampicillin/Sulbactam	4/23 (17.4)	40/76 (52.6)
Cefuroxime	8/23 (34.8)	21/76 (27.6)
Ampicillin/Sulbactam + Fosfomycin		1/76 (1.3)
Clindamycin	5/23 (21.7)	5/76 (6.6)
Penicillin	2/23 (8.7)	
Amoxicillin/Clavulanic Acid	2/23 (8.7)	2/76 (2.6)
Cefazolin		1/76 (1.3)
Daptomycin + Fosfomycin		1/76 (1.3)
Flucloxacillin		1/76 (1.3)
Vancomycin	2/23 (8.7)	2/76 (2.6)
Vancomycin + Fosfomycin		2/76 (2.6)

**Table 3:** List of bacteria identified in the cultures.

Bacteria	Occurrence in TSA, *n* (%)	Occurrence in TKA/UKA, *n* (%)	*p*-Value (*p* < 0.05 Is Significant)
**Gram positive**	**27 (100.0%)**	**78 (94.0%)**	***p* = 0.190**
**CPS**	**3 (11.1%)**	**20 (24.1%)**	***p* = 0.150**
*Staphylococcus aureus*	3 (11.1%)	19 (22.9%)	
*Staphylococcus aureus*, MRSA	0 (0.0%)	1 (1.2%)	
**CNS**	**16 (59.3)**	**36 (43.4%)**	***p* = 0.150**
*Staphylococcus capitis*	1 (3.7%)	3 (3.6%)	
*Staphylococcus epidermidis*	12 (44.4%)	21 (25.3%)	
*Staphylococcus hemolyticus*	0 (0.0)	4 (4.8%)	
*Staphylococcus hominis*	1 (3.7%)	3 (3.6%)	
*Staphylococcus lugdunensis*	0 (0.0%)	3 (3.6%)	
*Staphylococcus cohnii*	0 (0.0%)	1 (1.2%)	
*Staphylococcus warneri*	2 (7.5%)	1 (1.2%)	
***Streptococcus* spp.**	**0 (0.0%)**	**13 (15.7%)**	***p* = 0.029**
*Streptococcus agalactiae*	0 (0.0%)	6 (7.2%)	
*Streptococcus dysgalactiae*	0 (0.0%)	2 (2.4%)	
*Streptococcus oralis*	0 (0.0%)	1 (1.2%)	
*β-hemolytic streptococci group G*	0 (0.0%)	3 (3.6%)	
*Streptococcus mitis*	0 (0.0%)	1 (1.2%)	
**Anerobes**	**7 (25.9%)**	**3 (3.6%)**	***p* < 0.001**
*Cutibacterium acnes*	7 (25.9%)	0 (0.0%)	
*Peptoniphilus* sp.	0 (0.0%)	2 (2.4%)	
*Actinomyces turicensis*	0 (0.0%)	1 (1.2%)	
***Enterococcus* spp.**	**1 (3.7%)**	**6 (7.2%)**	***p* = 0.510**
*Enterococcus faecalis*	0 (0.0%)	4 (4.8%)	
*Enterococcus faecium*	1 (3.7%)	0 (0.0%)	
*Corynebacterium*	0 (0.0%)	2 (2.4%)	
**Gram negative**	**0 (0.0%)**	**5 (6.0%)**	***p* = 0.640**
*Enterobacter cloacae*	0 (0.0%)	3 (3.6%)	
*Escherichia coli*	0 (0.0%)	1 (1.2%)	
*Serratia marcescens*	0 (0.0%)	1 (1.2%)	
**Total number**	**27**	**83**	

CPS, coagulase-positive staphylococci; MRSA, methicillin-resistant Staphylococcus aureus; CNS, coagulase-negative staphylococci; n, number; TSA, total shoulder arthroplasty; TKA, total knee arthroplasty.

**Table 4:** Antibiotic resistances and their statistical significances in TSA and TKA revisions.

Type of Antibiotic	TSA (*n* = 27)		TKA (*n* = 83)		Sensitivity *p*-Value (*p* < 0.05 Is Significant)	Resistance *p*-Value (*p* < 0.05 Is Significant)
	S	R	S	R		
Ciprofloxacin	9/14 (64.3)	5/14 (35.7)	33/46 (71.7)	13/46 (28.3)	0.440	0.810
Clindamycin	18/26 (69.2)	8/26 (30.8)	43/66 (65.2)	23/66 (34.8)	0.270	0.960
Co-trimoxazole	17/18 (94.4)	1/18 (5.6)	41/45 (91.1)	4/45 (8.9)	0.320	0.770
Imipenem	2/2 (100.0)	0/2 (0.0)	9/13 (69.2)	4/13 (30.8)	0.560	0.230
Meropenem	4/4 (100.0)	0/4 (0.0)	6/6 (100.0)	0/6 (0.0)	0.270	n/a
Levofloxacin	7/15 (46.7)	8/15 (53.3)	28/50 (56.0)	22/50 (44.0)	0.360	0.860
Moxifloxacin	15/20 (75.0)	5/20 (25.0)	57/70 (81.4)	13/70 (18.6)	0.110	0.81
Oxacillin	12/19 (63.2)	7/19 (36.8)	37/53 (69.8)	16/53 (30.2)	0.830	0.540
Penicillin	7/19 (36.8)	12/19 (63.2)	28/60 (46.7)	32/60 (53.3)	0.360	0.720
Rifampicin	17/17 (100.0)	0/17 (0.0)	47/47 (100.0)	0/47 (0.0)	0.750	n/a
Tetracycline	17/17 (100.0)	0/17 (0.0)	49/53 (92.5)	4/53 (7.5)	0.930	0.230
Metronidazole	0/6 (0.0)	6/6 (100.0)	4/5 (80.0)	1 (20.0)	0.320	**<0.001**
Tigecycline	19/19 (100.0)	0/19 (0.0)	54/55 (98.2)	1/55 (1.8)	0.850	0.560
Ampicillin	0/2 (0.0)	2/2 (100.0)	8/16 (50.0)	8/16 (50.0)	0.085	0.680
Ampicillin + Sulbactam	9/10 (90.0)	1/10 (10.0)	17/30 (56.7)	13/30 (43.3)	0.220	0.091
Amoxicillin + Clavulanic acid	3/3 (100.0)	0/3 (0.0)	3/5 (60.0)	2/5 (40.0)	0.160	0.400
Piperacillin Tazobactam	7/8 (87.5)	1/8 (12.5)	12/13 (92.3)	1/13 (7.7)	0.210	0.420
Cefazolin	5/7 (71.4)	2/7 (28.6)	14/21 (66.7)	7/21 (33.3)	0.930	0.820
Cefuroxime	1/1 (100.0)	0/1 (0.0)	3/5 (60.0)	2/5 (40.0)	0.980	0.400
Erythromycin	7/19 (36.8)	12/19 (63.2)	42/66 (63.6)	22/66 (33.3)	**0.014**	0.110
Fosfomycin	18/19 (94.7)	1/19 (5.3)	44/56 (78.6)	12/56 (21.4)	0.320	0.120
Fusidic Acid	15/20 (75.5)	5/20 (25.0)	42/50 (84.0)	8/50 (16.0)	0.830	0.250
Linezolid	19/19 (100.0)	0/19 (0.0)	56/56 (100.0)	0/56 (0.0)	0.960	n/a
Teicoplanin	19/19 (100.0)	0/19 (0.0)	48/48 (100.0)	0/48 (0.0)	0.370	n/a
Vancomycin	19/19 (100.0)	0/19 (0.0)	49/49 (100.0)	0/49 (0.0)	0.440	n/a

TSA, total shoulder arthroplasty; TKA total knee arthroplasty; n, number; S, sensitive; R, resistant, n/a, not applicable.

**Table 5:** Accuracy of empirical antibiotics.

Empirical Antibiotic	Accuracy in TSA Revision Group—No. of Isolated Bacteria, n (%)	Accuracy in TKA/UKA Revision Group—No. of Isolated Bacteria, n (%)	*p*-Value (*p* < 0.05 Is Significant)
Ampicillin/Sulbactam	6/7 (85.7)	34/49 (69.4)	*p* = 0.370
Cefuroxime	6/9 (66.7)	18/21 (85.7)	*p* = 0.230
Ampicillin/Sulbactam + Fosfomycin		1/1 (100.0)	
Clindamycin	2/6 (33.3)	3/5 (60.0)	*p* = 0.080
Penicillin	3/3 (100.0)		
Cefazolin		1/1 (100.0)	
Daptomycin + Fosfomycin		1/1 (100.0)	
Flucloxacillin		1/1 (100.0)	
Vancomycin	2/2 (100.0)	2/2 (100.0)	
Vancomycin + Fosfomycin		2/2 (100.0)	
** Total accuracy **	** 19/27 (70.4) **	** 63/83 (75.9) **	***p* = 0.200**

**Table 6:** Preoperative antibiotics in shoulder arthroplasty revision.

Preoperative Antibiotic	Shoulder—No. of Patients, *n* (%)
Cefuroxime	20/23 (87.0)
Clindamycin	3/23 (13.0)

**Table 7:** Preoperative antibiotics in knee arthroplasty revision.

Preoperative Antibiotic	Knee—No. of Patients, *n* (%)
Cefuroxime	67/76 (88.2)
Clindamycin	7/76 (9.2)
Ampicillin/Sulbactam	1/76(1.3)
Vancomycin	1/76 (1.3)

## Data Availability

The data presented in this study are available upon request from the corresponding author.
